# Tumor-Derived Matrix Metalloproteinase-13 (MMP-13) Expression in Benign and Malignant Breast Lesions

**DOI:** 10.31557/APJCP.2021.22.8.2603

**Published:** 2021-08

**Authors:** Fereshteh Ameli, Firouzeh Ghafourian Nassab, Noraidah Masir, Farzan Khatib

**Affiliations:** 1 *Department of Pathology, Cancer Institute, Imam Khomeini Hospital Complex,Tehran University of Medical Sciences, Tehran, Iran. *; 2 *Department of Pathology, Faculty of Medicine, Universiti Kebangsaan Malaysia, Kuala Lumpur, Malaysia. *; 3 *Department of Electrical Engineering, Mashhad Branch, Islamic Azad University, Mashhad, Iran. *

**Keywords:** Matrix metalloproteinase 13 (MMP-13), breast cancer, prognostic marker

## Abstract

**Introduction:**

Breast carcinoma is the most common malignancy and the leading cause of cancer death in women. Matrix metalloproteinase-13 (MMP-13) is a hypothetical prognostic marker in invasive breast cancer. This study aimed to determine MMP-13 expression in benign and malignant breast lesions and to evaluate the correlation between MMP-13 expression and tumor characteristics in invasive ductal carcinoma (IDC).

**Materials and Method:**

We evaluated cytoplasmic expression of MMP-13 based on staining index using immunohistochemistry (IHC) in epithelial cells, stromal fibroblasts of IDC (n=90) and benign epithelial breast (n=90) lesions. Correlation between IHC and tumor size, lymph node status, distance metastasis, estrogen receptor (ER), progesterone receptor (PR) and Her-2/neu was assessed.

**Results:**

MMP-13 expression was 45% and 38.8% in malignant epithelial cells and peritumoral fibroblasts, respectively. Only low level of MMP-13 expression was seen in benign breast lesions (8.8% in epithelial component and 2.2% in stromal fibroblasts), while high level of MMP-13 expression was noted in malignant tumors, mainly grade II or III. Cytoplasmic MMP-13 expressions in epithelial tumor cells was correlated significantly with peritumoral fibroblasts. MMP-13 expression was directly correlated with distant metastasis and tumor stage in epithelial tumoral cells and was inversely correlated with progesterone expression in both tumoral and stromal cells.

**Conclusion:**

This study showed that MMP-13 was a moderator for tumor invasion and metastasis and could be an independent predictor of poor prognosis in breast cancer. The role of MMP-13 in predicting the risk of malignant transformation in benign lesions should be further investigated.

## Introduction

Breast cancer is still one of the most common causes of cancer-related deaths around the world (Sung et al., 2021). Despite significant advances in the diagnosis and treatment of breast cancer, predicting the aggressive behavior of the cancer, as well as detection and eradication of recurrences remained the greatest clinical challenge in oncology (Chang et al., 2009). Currently, the focus of cancer research is on understanding the underlying functional mechanisms in cell transformation and tumor progression that can be used to develop new markers and therapies (Quintero-Fabián et al., 2019). 

There is increasing evidence that breast cancer behavior is a product of interconnection between the malignant epithelial compartment and the surrounding microenvironment. In contrast to normal physiological stroma, where epithelial cell polarity is maintained and epithelial cell growth and transformation is inhibited, tumor stroma may stimulate the whole tumor development process (Folgueira et al., 2013).

Matrix metalloproteinases (MMPs) are a family of proteolytic enzymes, which are often calcium-dependent and contain zinc in their molecular structure. MMPs can degrade all kinds of extracellular proteins (Folgueira et al., 2013). At least two groups of extracellular proteins have been reported to play important roles in cancer metastasis and invasion. The first group includes some extracellular matrix components, including fibronectins, gelatins, and collagens, that could prevent cancer cells from moving freely into the nearby tissues, vessels and lymphatics. The second group includes some cell surface receptors, ligands, and chemokines and cytokines, which play an important role in cancer cell migration, proliferation or apoptosis through different cell signaling pathways (Wang et al., 2019).

Because of the abilities of MMPs in degrading these extracellular proteins, high expressions of MMPs have been widely reported to be related to cancer metastasis, progression, and poor prognosis (Wang et al., 2019). For example, MMP-19 is highly expressed in astroglial tumors and was found to be capable of facilitating the invasion of glioma cells through brain extracellular matrix components (Lettau et al., 2010). MMP-20 was found to have an important role in the progression of esophageal cancer (Yang et al., 2001). In addition, MMP-14 was reported to be associated with invasion and metastasis in ovarian carcinoma (Wang et al., 2019). 

MMP-13, also known as Human collagenase-3, is synthesized as a latent pro-enzyme and is activated by cleavage of the N-terminal peptide. Expression of MMP-13 has been detected in various types of cancers, including papillary thyroid carcinoma, colorectal, and breast cancers (Kotepui et al., 2016). Studies have further suggested that MMP-13 may play a central role in the extracellular MMP activation cascade, which results in degradation of the ECM network. It has been reported that the overexpression of MMP-13 was associated with invasive capacities of the malignant cells, including lymph node metastases, and shorter overall survival in several types of malignancies (Zhang et al., 2008; Kotepui et al., 2016). However, the clinical utility of MMP-13 as a breast cancer marker remains controversial.

The aim of this study was to evaluate the expression and prognostic value of MMP-13 tissue distribution pattern in human breast cancer compared to benign breast lesions. Furthermore, the correlation between MMP-13 expression and clinical parameters, including tumor size, histological grade, regional lymph node involvement, presence or absence of distance metastasis and tumor biomarkers was also assessed in this study.

## Materials and Methods

*Study population*

Invasive ductal breast cancer tissues that were diagnosed and surgically resected during 6 years at the Department of Pathology, University Kebangsaan Malaysia Medical Center were evaluated in this study. Study duration was determined based on the use of same clone of antibody (for breast biomarkers) on that duration. Tissues were excluded if prior radiotherapy or neoadjuvant therapies were performed before the recruitment of the tissues. Benign breast lesions, including fibrocystic changes, fibroadenoma, adenosis, papilloma and ductal hyperplasia with or without atypia, that were diagnosed by a pathologist were obtained from the same department. 

The clinicopathological variables including patients’ age at diagnosis, race, tumor size, tumor grade, regional lymph node involvement, and status of estrogen receptor (ER), progesterone receptor (PR) and Her-2/neu, as well as presence or absence of distance metastasis were obtained from corresponding histopathology reports via the Laboratory Information System and/or the surgical department records. All patients, unless deceased, were followed up for at least 48 months.

This study included a total of 90 cases of invasive ductal cancer (IDC) of the breast and 90 benign breast lesions. All slides from each case were reviewed by 2 independent observers (one pathologist and one trainee) and one representative slide was selected. The criteria for tumor grade, pathologic stage, scoring of ER, PR and Her-2/neu were obtained from College of American pathologists Cancer Protocol. The corresponding paraffin embedded tissue blocks of the selected slides were retrieved from the storage room of the department. This study was approved by the Ethics Committee of the University Kebangsaan Malaysia Medical Center. 

*IHC study*

Mouse monoclonal antibody MMP-13 (Collagenase-3) Ab-1 Clone VIIIA2, was used at a dilution of 1:25 for immunohistochemical (IHC) staining. The antibody was obtained from Thermo scientific (code no: LV-MS 825-P1) through Biomarketing Services.

IHC staining was performed on the whole section tissue slides. Staining was performed using the protocols from Dako REALTM EnvisionTM Detection System, Peroxidase/DAB+, Rabbit/Mouse (Code No.K5007). Four µm paraffin block sections were prepared from formalin-fixed, paraffin-embedded blocks and were taken on charged slides. Deparaffinization and rehydration was performed after 30-minute incubation at 60ºC on hot plate. The slides were subsequently incubated with hydrogen peroxide (H_2_O_2_) for 6 minutes followed by rinsing with running tap water. Antigen retrieval step was performed using heat-induced antigen retrieval pH 9.0 Tris-ethylenediaminetetracetic acid (EDTA)-based solution (DAKO) in the Pascal Pressurized Chamber (Dako Cytomation, USA) at 125°C for 30 seconds at the pressure of 20 psi followed by cooling at room temperature for 20 minutes and rinsing with running tap water. Slides were then incubated for 30 minutes at room temperature with primary antibody MMP-13 (dilution 1:25). Then, slides were incubated with Dako REALTM EnvisionTM/HRP, Rabbit/Mouse (ENV) for 30 minutes. Sections were then incubated with 1x DAB-containing Substrate Working Solution for 7 minutes. Hematoxylin counterstaining (30 seconds) was performed after the procedures were completed followed by dehydration, clearing and mounting steps using DPX mounting medium. Positive and negative controls were included with each batch of sections to confirm the consistency of the analysis. A case of IDC with strong cytoplasmic staining for MMP13 was used as positive control. For the negative control, the same sections were incubated omitting the primary antibody.

*Interpretation of the results*

IHC was scored independently using a semi-quantitative scoring system as described below. The intensity of the immunostaining was defined based on the negative and positive controls and was scored based on a 4-point score system. No brown particle staining: 0, light brown particle in cytoplasm: 1, moderate brown particle: 2, and dark brown particle; 3. The percentage of positive cells, as the determinant of the extent of immunostaining, was quantified under microscope using a 4-point score system. 1: <25% positive cells; 2: 25% to 50% positive cells; 3: 51% to 75% positive cells and 4: >75% positive cells. The staining index (SI), which is the product of the intensity and the percentage of positive staining, was used to define high (SI ≥ 6) or low (SI < 6) expression of MMP-13 (Zhang et al., 2008). Two pathologists, who were both blinded to patients’ clinicopathologic parameters and outcomes, performed the scoring.

*Statistical analysis*

Data analysis was performed using the statistical package for social sciences (SPSS) version 23 (Chicago, Illinois, USA). Continuous data was presented using mean and standard deviation (SD) and categorical data was presented using frequency and percentage. To test the potential value of MMP-13 as a breast cancer biomarker, chi-square, and fisher exact or Monte Carlo tests were conducted to evaluate the correlation of high and low level of MMP-13 expression with clinical and histopathological features (age, race, tumor size, tumor grade, lymph node status, metastasis, tumor stage and ER, PR and HER2/neu expression). P values of less than 0.05 were considered statistically significant.

## Results

This study included a total of 90 cases of IDC and 90 benign breast tissues. The age of the patients ranged from 19 to 80 years (mean age was 50.47 years). The youngest patient was diagnosed with fibroadenoma and the oldest one was diagnosed with IDC. Age at diagnosis for benign breast lesions ranged from 19 to 72 years old (mean age was 45.34 years). Majority of benign cases were fibrocystic. Age at diagnosis for malignant cases ranged from 23 to 80 years old (mean age was 55.59 years). Majority of the study patients were Malay (103, 57.2%), followed by Chinese (53, 29.4%), Indian (14, 7.8%) and other ethnicities (10, 5.6%). 

Most of the tumors in invasive breast carcinoma (58, 64.4%) were 2-5 cm in size and 53 (58.9%) patients had axillary lymph node involvement. Tumor grade 2 was the most common (41, 45.6%), followed by grade 1 (26, 28.9%) and grade 3 (23, 25.6%). Based on the archive of the pathology reports, ER was positive in 58 (64.4%) and negative in 32 (35.6%) patients, whereas PR was positive in 59 (65.6%) and negative in 31 (34.4%) patients. Her-2/neu expression was negative in 56 (62.2%) and positive in 34 (37.8%) patients. There were distant metastases in 24 (26.7 %) patients, while no distant metastases were seen in 66 (73.3%) patients after four years of follow up. The most common site of metastases was bone (11/24, 12.2 %) followed by liver (2/24, 2.2%), lung, brain and mediastinal lymph node (1/24, 1.1% for each). There were 8 (8.9%) unknown sites of metastasis. 

According to the pathological TNM classification and stage grouping of the breast cancer, 31.1% (28/90) of the patients were in stage IIA, 21.1% (19/90) were in stage IIB and 26.7% (24/90) were in stage IV. Among stage III patients, 14.4% (13/90) were in stage IIIA and only 2.2% (2/90) were in stage IIIC. However, none of the patients were in stage IIIB. Finally, only 4.4% (4/90) of the patients were in stage IA (Table 1). 

**Figure 1:**
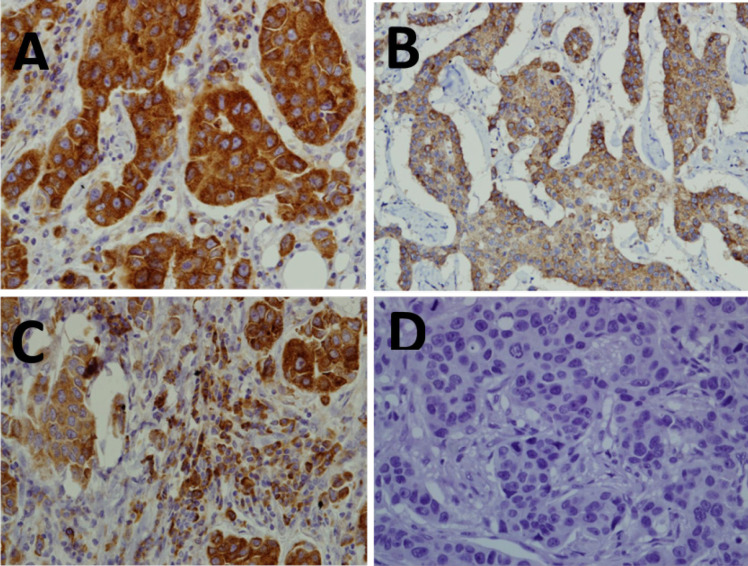
Differential Immunoreactivity of MMP-13 in Breast Cancer and Peritumoral Fibroblast Cells. (A): High level of MMP-13 expression in cancer cells of IDC (X200). (B): Low level of MMP-13 expression in cancer cells of IDC (X200). (C): High level of MMP-13 expression in both the cancer cells and peritumoral fibroblast cells of IDC(X200). (D): Infiltrative ductal carcinoma with negative MMP-13 expression(X200). The brown color represents the IHC staining of MMP-13. The blue color represents the nuclear counterstain

**Figure 2:**
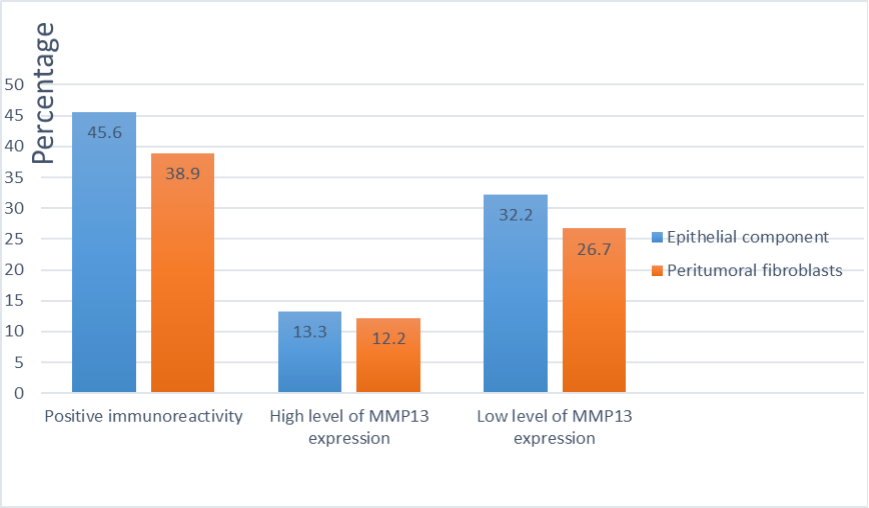
Distribution of MMP13 Expression in Infiltrative Ductal Carcinoma Cases

Among IDC patients, MMP-13 expression was detected in cytoplasm of malignant cells and peritumoural fibroblasts in 45.5 % (41/90) and 38.9% (35/90) of the patients, respectively. The SI showed high level of MMP13 expression in 29.3% (12/41) of patients with IDC, whereas 70.7% (29/41) of the IDC patients showed low level of expression (Figure 2). High level of MMP13 expression was observed in 31.4% of IDC patients with positive peritumoral fibroblasts (11/35), while 68.6% (24/35) showed low level of MMP-13 expression (Figure 1). 32% (29/90) of malignant cases show expression of MMP13 in both epithelial and stromal fibroblasts including 10 high SI and 12 low SI in both component. Seven positive cases show discrepancy of expression (low and high SI) between epithelial and stromal fibroblasts. 

MMP13 was positive in epithelial cells of 8.8 % (8/90) of benign breast lesions and in 2.2% (2/90) of stromal fibroblasts. However, MMP-13 displayed only low level of expression in all positive benign breast lesions. Overall, the detection of cytoplasmic MMP-13 in epithelial malignant cells, correlated significantly with the cytoplasmic MMP-3 in the peritumoral fibroblasts (p < 0.001) (Figure 2). There was a significant difference between benign and malignant breast lesions in expression of MMP-13 (p=0.001).

High levels of MMP-13 expression correlated with tumor stage and distant metastases only in cancer cells (p < 0.001 and p < 0.002, respectively). PR expression was also inversely correlated with MMP-13 expression in both tumoral cells (p<0.001) and stromal cells (p = 0.020). This finding indicated that PR positivity reduced with increased MMP-13 expression. However, no significant association including low or high staining index was found between MMP-13 expression tumor size, lymph node involvement, histological grade, ER, and HER2neu. 

MMP-13 expression was not correlated with age nor race of the patients (Table 2). There was a significant statistical correlation between distant metastasis with tumor staging (p=0.001) and axillary lymph node status (p = 0.001). No statistically significant correlation was seen between tumor grade (p = 0.24), tumor biomarkers (ER: p= 0.47; PR: p=0.38; c-erbB2: p=0.28) and patient age (p = 0.92) with distant metastases. 

**Table 1:** Demographics of Patient and Tumor Characteristics of Invasive Ductal Carcinoma

Parameters/markers		Number of cases	Percent
Age(years)(mean55.5)	<40	7	7.8
	40-50	22	24.4
	50-60	29	32.2
	>60	32	35.6
Race	Malay	54	60.0
	Chinese	29	32.2
	Indian	3	3.3
	Others	4	4.4
Tumor size	<2	13	14.4
	2-5	58	64.4
	>5	19	21.1
Tumor Grade	1	26	28.9
	2	41	45.6
	3	23	25.6
Lymph node status	Negative	37	41.1
	Positive	53	58.9
Estrogen Receptor	Negative	32	35.6
	Positive	58	64.4
Progesterone Receptor	Negative	31	34.4
	Positive	59	65.6
Her-2/neu expression	Negative	56	62.2
	Positive	34	37.8
Distant Metastasis	Absent	66	73.3
Site of metastasis	Present	24	26.7
	Bone	11	12.2
	Brain	1	1.1
	Liver	2	2.2
	Lung	1	1.1
	Mediastinal LN	1	1.1
	Unknown	8	8.9
Staging	IA	4	4.4
	IIA	28	31.1
	IIB	19	21.1
	IIIA	13	14.4
	IIIB	0	0.0
	IIIC	2	2.2
	IV	24	26.7
Expression of	Negative	49	54.4
MMP-13 in malignant			
epithelial cells Expression of MMP-13	Positive	41	45.6
	Negative	55	61.1
in stromal cells	Positive	35	38.9
	Total	90	100.0

**Table 2:** Expression of MMP-13 in Malignant Epithelial and Stromal Fibroblast Cells in Invasive Ductal Carcinoma Based on Staining Index (SI)

	Total n	High expression of MMP-13 n (%)	Low expression of MMP-13 n (%)
Malignant cells	41	12 (29.3%)	29(70.7%)
Stromal fibroblasts	35	11(31.4%)	24(68.6%)

Low expression: SI <6 High expression: SI >= 6

**Table 3:** Correlation of High MMP-13 Expression with Clinicopathological Parameters and Other Biomarkers

Parameters/markers		Total	Negative result	Cancer epithelial cells			Peritumoral fibroblasts		
		N48= (%)	N=48 (%)	High SI	Low SI	P-value	High SI	Low SI	p-value
				N=13 (%)	N=29 (%)		N=11 (%)	N=24 (%)	
Age group	<40	7 (7.8)	2 (28.6)	0 (0.0)	5 (17.2)	0.056	0 (0.0)	1 (4.5)	0.544
	40-50	26 (28.9)	18 (37.5)	4 (30.8)	4 (13.8)		5 (33.3)	5 (22.7)	
	50-60	28 (31.1)	10 (20.8)	5 (38.5)	13 (44.8)		7 (46.7)	7 (46.7)	
	>60	29 (32.2)	18 (37.5)	4 (30.8)	7 (24.1)		3 (20.0)	9 (40.9)	
Race	Malay	54 (60)	31 (64.6)	7 (13)	17 (31.5)	0.678	5 (9.3)	15 (27.8)	0.6
	Chinese	29 (32.2)	14 (29.2)	4 (13.8)	11 (37.9)		5 (17.2)	8 (27.6)	
	Indian	3 (3.3)	1 (2.1)	1 (33.3)	0 (0)		1 (33.3)	0 (0)	
	Others	4 (4.4)	2 (4.2)	0 (0)	1 (25)		0 (0)	1 (25)	
Tumor size	<2	13 (14.4)	11 (22.9)	2 (15.4)	4 (30.8)	0.889	3 (23.1)	2 (15.4)	0.522
	5-Feb	58 (64.4)	28 (58.3)	7 (12.1)	20 (34.5)		6 (10.3)	19 (32.8)	
	>5	19 (21.1)	9 (18.8)	3 (15.8)	5 (26.3)		2 (10.5)	3 (15.8)	
Grade	G1	26 (28.9)	17 (35.4)	3 (11.5)	6 (23.1)	0.243	3 (11.5)	5 (19.2)	0.211
	G2	41 (45.6)	23 (47.9)	4 (9.8)	13 (31.7)		4 (9.8)	9 (22)	
	G3	23 (25.6)	8 (16.7)	5 (21.7)	10 (43.5)		4 (17.4)	10 (43.5)	
LN	Negative	37 (41.1)	21 (43.8)	3 (8.1)	14 (37.8)	0.278	5 (13.5)	10 (27)	0.845
	Positive	53 (58.9)	27 (56.3)	9 (17)	15 (28.3)		6 (11.3)	14 (26.4)	
Metastasis	Negative	66 (73.3)	40 (83.3)	6 (9.1)	21 (31.8)	0.002	6 (9.1)	18 (27.3)	0.072
	Positive	24 (26.7)	8 (16.7)	6 (25)	8 (33.3)		5 (20.8)	6 (25)	
Staging	Early (I,II)	51 (56.7)	27 (56.3)	5 (9.8)	20 (39.2)	0.001	5 (9.8)	16 (31.4)	0.472
	Late (III,IV)	39 (43.3)	21 (43.8)	7 (17.9)	9 (32.1)		6 (15.4)	8 (20.5)	
ER	Negative	32 (35.6)	12 (25)	5 (15.6)	12 (37.5)	0.072	5 (15.6)	10 (31.3)	0.331
	Positive	58 (64.4)	36 (75)	6 (10.3)	17 (29.3)		6 (10.3)	14 (24.1)	
PR	Negative	31 (34.4)	9 (18.8)	8 (25.8)	13 (41.9)	0.001	6 (19.4)	12 (38.7)	0.02
	Positive	59 (65.6)	39 (81.3)	4 (6.4)	16 (27.1)		5 (8.5)	12 (20.3)	
HER2/neu	Negative	56 (62.2)	43 (89.6)	5 (8.9)	18 (32.1)	0.567	6 (10.7)	16 (28.6)	0.144
	Positive	34 (37.8)	5 (10.4)	7 (20.6)	11 (32.4)		5 (14.7)	8 (23.5)	

## Discussion

Invasion and metastasis are the results of complex interactions between cancer cells and normal stroma and are the major causes of cancer related morbidity and mortality. Invasion to the extracellular matrix (ECM) and vascular dissemination are two important phases of the metastatic cascade (Kumar et al., 2014). Different families of proteases, including MMPs, cathepsin D, and urokinase plasminogen activator, have been implicated in tumor cell invasion. It is evident that the proportion of MMPs and other macromolecules (cytokines, growth factors, and fibers, including integrins, polysaccharides) in the ECMs of tissues are metabolically, microenvironmentally, and epigenetically balanced for their functions in normal conditions (Quintero-Fabián et al., 2019).

The human MMPs family has 24 members that function as proteolytic enzymes and can dissolve extracellular matrix. This family of proteins not only participates in normal physiological functions, including wound healing and modulating growth factors and enzyme activation pathways, but also plays an important role in cancer infiltration, metastasis and angiogenesis (Chang et al., 2009). MMP-13 (Collagenase-3) is the latest human collagenase described in literature (Zakaria et al., 2019). MMP-13 is expressed in a broad range of primary malignant tumors and has been emerged as a novel biomarker (Pivetta et al., 2011). MMP-13 (collagenase-3) has important roles in cancer invasion, metastasis, growth regulation, immune evasion, apoptosis, and angiogenesis. Nielsen et al. pointed out that MMP13 breaks down basement membranes of tissues to form an invasive cancer in the process of breast cancer transition from ductal carcinoma in situ to invasive ductal carcinoma (Zakaria et al., 2019).

Elevated levels of MMP13 have been associated with decreased overall survival and increased lymph node metastasis in breast cancer (Zhang et al., 2008). Recently, Sung et al. highlighted a positive correlation between Gremlin-1 (GREM1) and MMP13 expression in breast cancer patients (Sung et al., 2020). They reported that GREM1 activated signal transducer and activator of transcription 3 (STAT3) transcription factor involved in the expression of MMP13. GREM1 can also promote lung metastasis in breast cancer cells through the STAT3-MMP13 pathway (Sung et al., 2020).

Wang et al. showed that high mRNA expression of MMP-19 and MMP-20 were independent predictors of poor outcome and resistance to several anti-cancer drugs in patients with ovarian serous carcinoma (Wang et al., 2019).

This study was conducted to investigate the expression of MMP-13 in benign and malignant breast lesions and to investigate the correlation between MMP-13 expression and clinicopathological parameters in IDC. The results of this study revealed that MMP-13 protein expression level in breast cancer tissues was significantly higher than benign breast tissues. This finding represents the role of MMP-13 in the tumorigenesis and progression of human breast cancer. 

Increased number of involved lymph nodes, metastasis and, as a result, later stage of tumor in patients with high level of MMP-13 expression suggests that MMP-13 probably plays a role in promoting tumor invasion and metastasis. These findings are consistent with other studies in breast cancer (Uría et al., 1997; Nielsen et al., 2001; Quintero-Fabián et al., 2019; Sung et al., 2021) as well as other tumors, including esophageal squamous cell carcinoma (Sedighi et al., 2016), malignant melanoma (Corte et al., 2005), gastric cancer (Huang et al., 2005) and papillary thyroid carcinoma (Wang et al., 2013). 

Furthermore, our study showed that 8.8% (8/90) of benign breast lesions expressed low level of MMP-13 in epithelial cells. This expression was observed in 5 fibrocystic lesions, 2 papilloma and one adenosis lesions. Low level MMP-13 expression was observed in 2.2% (2/90) of stromal lesions. 

Our study did not show high level expression of MMP13 in benign breast lesions. Corte et al. showed that benign melanocytic lesions were consistently negative for MMP-13 (0/8) (Corte et al., 2005). Zyada and Shamaa (2008) showed no positive staining for MMP-13 observed in normal cartilage tissue (0/2). Similarly, no MMP-13 expression has been reported in normal mammary gland and benign mammary lesion (0/9) (Heppner et al., 1996) and in several normal tissues including uterus, liver, prostate, parotid gland, breast, fibroadenomas (Freije et al., 1994). On the other hand, it seems that MMP-13 is not unregulated as a mediator of ECM breakdown in benign breast lesions and other normal tissues.

Zhang et al., (2008) showed that high levels of MMP-13 in cancer cells and peritumoral fibroblasts correlated with the expression of the Her-2/neu protein. In our study we could not find any significant correlation between MMP-13 expression in cancer cells and peritumoral fibroblasts and expression of the Her-2/neu protein and ER. However, this study showed that PR positivity reduced with increased MMP-13 expression, which needs to be investigated in further research. 

Both MMP-2 and MMP-9 have been extensively studied as biomarkers and therapeutic targets in different types of cancers (Zhang et al., 2008; Roy et al., 2009; Oguić et al., 2014; Webb et al., 2017; Wang et al., 2019). Since MMP-13 is also a metalloproteinase, it may act in a similar manner as MMP-2 and MMP-9. Thus, the value of abnormal MMP-13 expression in cancer tissues and its role as a prognostic marker or therapeutic target could further be evaluated. 

Conclusively, on the basis of positive expression of MMP-13 in majority of high-grade malignant tumors and low expression of detectable MMP-13 in few benign lesions, a possible role can be proposed for MMP-13 in the tumoral process and metastasis. It will be of interest to examine whether the expression of MMP-13 in a small percentage of benign lesions could be indicative of an increased risk of malignant transformation in these patients.

## Author Contribution Statement

The authors confirm contribution to the paper as follows: study conception and design: Firouzeh Ghaourian Nassab; data collection: Firouzeh Ghaourian Nassab; analysis and interpretation of results: Farzan Khatib and Fereshteh Ameli; draft manuscript preparation: Fereshteh Ameli and Firouzeh Ghaourian Nassab; Supervision of this research and findings of this work: Noraidah Masir. All authors discussed the results and contributed to the final manuscript.
